# Optimal placement of distributed generation to minimize power loss and improve voltage stability

**DOI:** 10.1016/j.heliyon.2024.e39298

**Published:** 2024-10-12

**Authors:** Samson Ademola Adegoke, Yanxia Sun, Adesola Sunday Adegoke, Damilola Ojeniyi

**Affiliations:** a Department of Electrical and Electronic Engineering Science, University of Johannesburg, South Africa; b Department of Electrical and Electronic Engineering, Ladoke Akintola University of Technology, Ogbomoso, Oyo State, Nigeria

**Keywords:** DG, Voltage stability, Diminishing power and energy loss, Voltage profile improvement, Metaheuristic techniques

## Abstract

Voltage instability is a major problem facing power utility companies due to a lack of maintenance and financial support to maintain the existing power system networks (PSN). Lack of maintenance occurs when PS fails to replace the aging equipment and services and upgrades existing networks. Also, the lack of financial support occurs when there is insufficient financial capital to carry out the above maintenance activities, which has incurred greater power loss in the systems. There is a need to eliminate the loss incurred in the system to avoid voltage collapse. The best way to increase the lifespan of a PSN and improve voltage stability is the optimum allocation of distributed generation (DG). The most common DG are solar photovoltaic (PV) and wind turbines. This review discusses the economic, environmental, and technical benefits of non-traditional DG technologies over a traditional system. However, this work reviewed different DG technologies that can be incorporated into PSN for better loss reduction. Detailed descriptions of energy loss, power loss, and multi-objectives for optimal DG planning were also discussed, along with comprehensive methods used in the sizing and placement of DG for voltage profile improvement (VPI). The findings recommend that the hybrid meta-heuristic algorithm be used for DG placement has it significantly improves voltage profile and lowers power loss. The present study concluded that with proper DG placement, the advantage of DG can be maximally accomplished, the voltage profile is improved, and losses are minimized.

## Abbreviation

ABC: Artificial bee colony
AM: Analytical method
BA: Bat algorithm
BHO: Black hole optimization
BSA: Black-tracking search algorithm
CBGA: Chu-Beasley genetic algorithm
CIGRE: The International Council on Large Electric Systems
DEA: Differential evolution algorithm
EP: Evolutionary programming
FA: Firefly algorithm
GA: Genetic algorithm
HSA: harmony search algorithm
HGPSO: Hybrid genetic particle swarm optimization
HSDO: Harmony search algorithm differential operator
IWD: Intelligent water drop
LSF: Loss sensitivity factor
LSI: Loss sensitivity index
MINLP: Mixed integer non-programming
MPSO: Multi-objective particle swarm optimization
MMPO: Modified marine predator optimization
OPF: Optimal power flow
PSO: Particle swarm optimization
QOSIMBO-Q: Quasi-Oppositional Swine Influenza Model-Based Optimization with Quarantine
REPSO: Rank evolutionary particle swarm optimization
SCA: Sine cosine algorithm
SOCP: Second-order cone programming
SPSO: Selective particle swarm optimization
TLBOA: Teaching learning-based optimization algorithm
VSA: Vortex search algorithm

## 1 Introduction

### 1.1 Background

**Fig. 1:**
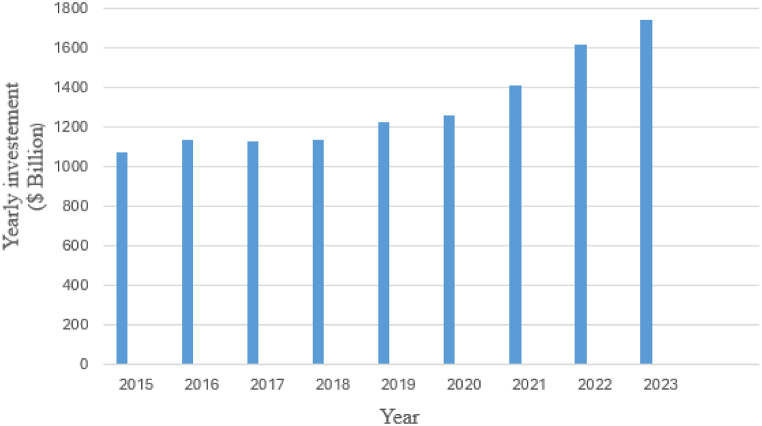
Yearly investments in DG technologies worldwide [3].

Due to the high pollutants the conventional electrical generating system releases into the atmosphere, electrical utilities have shifted to distributed generation (DG) to generate electricity since it gives clean energy, improves the voltage profile, and greatly reduces power loss. DG is among the various technologies that generate electricity far or near the load, such as photovoltaic (PV), wind, combined heat, power (CHP), etc. In 2020, Denmark supplied more than 50 % of its electricity from DG (solar PV and wind). Also, DG provided 30 % of its electricity in Ireland in 2020. In 2050, the United States and China were planning to generate electricity from DG of about 80 % and 60 % [1]. In 2022, the global market of DG has been estimated to be US $245.8 billion, and it is expected to reach US $615.9 billion in 2030 [2]. Global clean energy has experienced dynamic growth. In 2023, the global investment of DG has grown to US$1740 billion [3]. As illustrated in Fig. 1, the yearly investments in DG have significantly improved in 2023 due to several advantages. DG can serve a single building and office, and it could also serve industry, school, and military stations. DG gives reliable and clean energy to the end end-user. The familiar DG used in residential sectors is solar PV, small wind turbines, and natural gas-fired fuel cells. Also, in the commercial and industrial sectors, there are CHP, solar PV, wind, and biomass.

Power system (PS) utilities face many challenges due to rapid load increases and insufficient reactive support in transmission networks. Moreover, the inadequate support of reactive power is a problem in distribution systems. Today, most PS company generators operate under stressed conditions and are close to the operating limits due to strict economic constraints [4]. The existing transmission network was affected due to a lack of maintenance, making it operate close to operating limits [5]. There are wide ranges of blackouts globally, such as in Egypt, Sweden, Greece, Belgium, Tennessee, the USA, and others [[6], [7], [8], [9]]. Voltage collapse is a significant cause of blackouts in countries like Italy, Southern Finland, North America, London, Sweden, Tokyo, Canada, etc. [5,10]. Voltage stability-based DG placement improved the power grid's resilience against any unwanted event that could cause blackouts and voltage stability problems [11]. There is a need to invest in the system's transmission to increase capacity or provide end-user demand locally by DG. Traditional generators are gas turbines and diesel generators, and non-traditional generators are green energy (PV), wind turbines, fuel cells, storage devices, etc. [[12], [13], [14]]. DG may affect the voltage profile and power conditions of the system either positively or negatively [15]. The positive impact is the system support benefit, including loss reduction, voltage support, system utility reliability, and power quality. However, the benefit will only be achieved when DG is adequately sized and placed in proper locations [16,17]. When the power losses in distribution and transmission networks have increased, the existing system's efficiency is reduced. Shunt capacitor installation in distribution systems has been reported to minimize actual power loss effectively [[18], [19], [20]]. Power losses in distribution are owed to the energy loss when heating a conducting material (Joule effect), which accounts for 13 % of the generated energy [4].

**Fig. 2:**
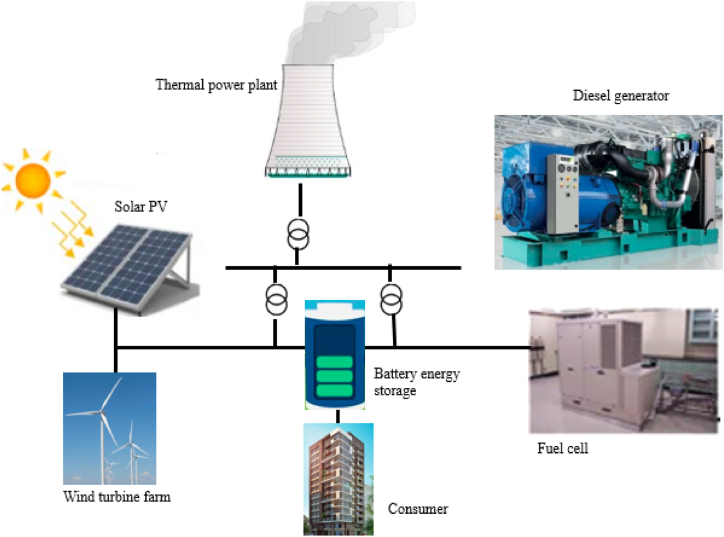
Power system in the presence of DG.

However, distribution companies keep actual losses below the acceptable value to make a profit [21]. Much research is on the distribution system's real power loss minimization. Adequate reactive support is required to keep the voltage stability and reliability of the system. Therefore, an innovative technology solution for the optimum utilization of traditional resources and resolution to satisfy consumer requirements with cost-effective power quality is needed. To tackle the challenges in an economical and proper time is the optimum allocation of DG (OADG) [4]. DG has numerous merits over traditional power generation, such as incremental voltage profile, system stability improvement, transmission and distribution congestion, pollutant reduction, and power loss reduction. Nevertheless, after the PS had been deregulated, some non-utilities companies shifted to small or large solar PV, microturbine, wind, etc., or a combination of two to meet the active power demand and gain profit [22,23]. A typical layout of a power system in the presence of DG is given in Fig. 2.

The issues of power loss and voltage stability improvement have raised great concern for researchers to use different methods such as metaheuristic, traditional, and analytical methods. Analytical and traditional methods suffer some difficulties, so metaheuristics have been used to overcome the challenges and handle complex issues in single and multiobjectives. Adeleke et al. [24] have reported the impact of DG in DNS to minimize greenhouse emissions (GHE), power loss, and enhanced electricity supply [24]. Harris Hawk Optimization (HHO) and Teaching Learning-Based Optimization (TLBO) have been reported to reduce GHE, power loss, yearly energy saving, and improve voltage profile [25]. Different types of DGs were used, and the result revealed that HHO and TLBO were sufficient to lower the losses and improve voltage better than the rest of the algorithms [25]. The optimum allocation of DG to improve the distribution performance using PSO was reported to minimize power loss, lower power interruption, and improve voltage profile [26]. Multiple PV-DG units have been reported for voltage profile improvement and power loss reduction using HGWO-PSO [27]. The results reveal that HGWO-PSO gives a better result than the rest of the methods in the literature [27]. Modified GWO was reported to allocate DG in DN, minimize total power loss, and improve voltage profile while maintaining constraints [28]. The Hummingbird Optimization Algorithm (HOA) was reported to minimize voltage deviation and power loss for single and multi-objective functions [29]. DG was used to minimize power losses and enhance the voltage profile using GA. Different loads have been considered on distribution lines using GA [30]. Archimedes optimization algorithm (AOA) was reported for the location of solar PV to minimize GHE and network dependence [31].

The placement of DG in the PS minimizes power loss in the network and gives a better voltage profile [32]. Moreover, the piercing level of DG and power losses give a U-trajectory. However, the absence of DG may lead to a rise in the power loss in the network and reduce the voltage profile beyond acceptable limits. Electrical utility companies encounter technical and non-technical issues, so this problem will not be allowed. The total power losses in the system were reduced by placing DG to improve the voltage profile. DG placement has been considered a real energy source in the past decade, and reactive power control improves the voltage profile and reduces power losses [16,33,34]. DG's influence on the distribution system's load-ability has been found in much literature. It was reported that there is an increase in distribution system load-ability with DG placement [35,36]. Different methods have been used to boost the system load-ability by improving system voltages profile [37,38]. Also, identifying the weakest bus in the system is an essential factor in increasing the system's load-ability, and the selected weakest bus is where DG is to be placed. The modal analysis was reported to determine the optimum placement of DG. The continuation power flow (CPF) method was used to determine the weakest bus and for optimum placement of DG, and it's further used for multi-DG placement [39,40]. An active distribution network (ADN) was reported to increase the voltage stability load-ability limits. However, control and regulation improved the power network with renewable energy (RE) units [36]. A marine predator algorithm (MPA) was used for multi-dimensional uncertain renewable DG [41]. Therefore, DG placement is necessary for higher benefits with little investment cost [42,43]. Table 1 shows the overall losses in distribution and transmission in different countries.

**Table 1:** Overall losses in distribution and transmission in different countries in the percentage of output [44].

S/N	Countries	Percentage (%) of total output power
1	Indian	21.1 %
2	Pakistan	16.9 %
3	Developing countries (UN classification)	16.1 %
4	Iran	14.6 %
5	Balagedesh	10.3 %
6	Russia Federation	10 %
7	Saudi Arabia	9.4 %
8	World average value	8.1 %
9	United Kingdom	7.6 %
10	Malaysia	6.4 %
11	United State	6 %
12	Australia	5.3 %
13	Germany	4.3 %

### 1.2 Research gap and contribution of the work

In the past decade, DG has witnessed remarkable attention from various researchers, and different reports have been reported to understand the research trend. For instance, some reviews on DG planning have been reported [[45], [46], [47], [48]]. Computing, analytical, MINLP, 2/3 rule, and OPF methods for distributing RE on distribution systems were reported [45]. DG's impact, definition, status of DG technologies, and optimization methods used in DG planning in distribution systems have been presented [46]. A bibliographic survey on loss minimization methods used in DN, like DG allocation, network reconfiguration, and placement of capacitors, has been documented [47]. In addition, a review of different methods (classic, metaheuristic, and hybrid) for allocating DG in distribution systems has been reported [48]. A critical review of strategies for simultaneous DG and capacitor bank (CB) placement in electrical DN has been reported [49]. Interestingly, multiobjective, multiple constraints, and optimization-based models using computing methods in DG planning have been reported [50,51]. Another author presented DG units and DN reconfiguration planning using three-dimensional group search optimization (3DGSO) [52]. Ant colony optimization (ACO) was reported to minimize power loss and enhance voltage profile by sizing and location of DG [53]. Improved HHO has been reported to solve single and multi-objectives by allocating DGs in DN [54]. Transient search optimization (TSO) was reported for multiple locations of DG in radial DN, and the result was superior to other methods reported in the literature [55]. Sine cosine optimization has been reported for the allocation of DG to improve voltage profile and power loss minimization [56]. A pathfinder algorithm has been reported for the location of solar PV in multi-lateral DN to improve the system resilience [57].

All the works mentioned above only discussed the various optimization techniques used in the allocation and size of DG, the definition of DG, and loss minimization. To the best of our knowledge, the critical discussion and comprehensive review of the various DG technologies have not yet been presented, which is in addition to the current trend that has not been covered in this field. Therefore, this review is imperative to provide up-to-date research trends in the field. This enables the present study to discuss the strengths and weaknesses of the technologies available and provides some insights for further investigation. It discusses different DG technologies, the benefits of DG over traditional power systems, and multi-objective (MO) optimization for optimal DG planning. In addition, techniques and software used for DG placement, such as conventional and optimization algorithms (single and hybrid) for optimal placement for voltage stability improvement, are reviewed. The data used were sources from Google Scholar, Scopus®, Elsevier, IEEE Explore, Springer, MDPI, IET, Taylor & Francis, and the Web of Science database using the keywords minimization of power and energy loss, voltage profile improvement, optimal allocation and sizing of distributed generation. Only the articles written in English language were considered. Therefore, the rest of this paper is structured as follows: Section 2 provides various DG definitions and technologies. Section 3 gives the impact of DG on voltage stability. Section 4 discusses the optimal planning of DG in the distribution system to minimize losses. Section 5 discusses voltage stability improvement-based DG for optimal planning in the distribution system**.** Section 6 discussed multi-objective (MO) optimization for optimal DG planning. Section 7 gives methods used in DG placement for voltage stability improvement**,** and the last Section 8 gives the conclusion and future work.

## 2 Distributed generation (DG)

Due to the impact of DG on improving voltage profile and giving room for expansion as the load grows, many researchers have worked on DG potential to minimize losses and meet the load demand. DG also offers economic, technical, and environmental benefits. However, much work is needed on DG to reduce the losses using metaheuristics. Still, some challenges are not met, which require a future perspective, such as the reduction in harmonic pollution and frequency stability with DG, e.g., energy storage technologies to explore more potential of DG, etc., which are not presented.

Depending on the countries and different agencies, there are various ways of defining DG. According to the International Energy Agency (IEA), DG is a generating plant serving consumers on-site or providing support for distribution networks connected to the grid at the distribution level. However, it's an electric power source connected straight to the end-user side meter or DN. Another agency, CIGRE, further defines DG as a generation station that has the following futures: a) it is usually connected to distribution networks, b) it is not centrally dispatched, c) it is not centrally planned, and it is in small portion range between 50 and 100 MW. Furthermore, the EPRI defines DG as a generation that ranges from little kilowatts to 50 MW. Therefore, DG is a small-scale generation [58,59].

DG's general definition and relevant issues in the competitive electrical market were reported [60]. Also, the authors reported connection and network issues of DG along with distributed utility, distributed resources, and distributed capacity [60]. The difficulties encountered by DG protection (such as false tripping, blinding protection, unsynchronized reclosing, variation in short circuit levels, etc.) are discussed. Also, the challenging issues in a microgrid (such as islanding, power imbalance, voltage and frequency control, etc.) are discussed. Moreso, the possible solution is to guide the future of active distribution networks (e.g., symmetrical and differential current components, balanced combination of different sources of DG types, fault current limiters, etc.) provided in the literature [61]. Shuaibu Hassan et al. reported the significance of placing DG in the exact location to achieve the full benefits (e.g., technological, environmental, and economic). Otherwise, the energy quality and operation may be altered. The various prospective paths to increase the multi-approach that have not been established are discussed from the meta-heuristic optimization point of view [59]. Sultana et al. present a comprehensive study on optimal DG placement for power loss, energy loss, voltage stability improvement, and voltage profile enhancement [4]. The authors also present a detailed explanation that will be useful for power system planning to know the objective and planning factors that need proper attention for the optimal location of DG [4]. DG ranges from micro-distributing generation up to large distributed generation. Table 2. Shows different sizes of DG technology.

**Table 2:** Different types and sizes of DG technology [48,60,62].

DG type	Technology	Size
Micro-DG	Solar PV	Wattages to 5 kW (1W -5 KW)
Small-DG	Wind turbine, biomass, Fuel cell.	Five kilowatts to 5-MW (5KW-5MW),
Medium-DG	Geothermal	Five-megawatt to 50 MW (5MW–50MW)
Large-DG	Hydrogen energy system	Fifty megawatts and three hundred megawatts (50MW–300MW)

Due to technological advances, several DG are available, and many are in the research and development stage. Some well-known RES are solar PV, wind turbines, and biomass; each has its characteristics and advantages. Of all the DG available, the most installed are diesel engines, gas engines, and gas turbines. However, the older diesel engine has been improved, and new DG, such as microturbines, has been introduced. The fuel cell is the future technology that researchers need to work on. Some prototype demonstrations have been available. The installation cost of green energy is expected to reduce over the next decade.

The primary application of DG is to provide a friendly environment through green energy (renewable energy), reduce the cost of electricity and CHP, provide good reliability and quality of supply power, and defer the distribution and transmission line investment through improved load-ability. Also, the application of DG in a deregulated environment is in the form of ancillary services, including the spinning and non-spinning reverse, voltage control, and reactive power supply. Some other benefits of DG are reducing energy costs through CHP generation, less exposure to price volatility, and avoiding electricity transmission costs.

DG has been considered a solution to most of the problems facing utilities today. Several issues, such as tripping off lines, generator loss, changes in the protection scheme, etc., need to be resolved. However, the type of DG adopted will determine the solution [[63], [64], [65]].

### 2.1 Technologies of DG

DG can exist in two forms: traditional and untraditional energy systems. Traditional DG energy resources are microturbines, petrol or diesel generators, and Stirling engines. At the same time, the non-traditional DG energy resources are wind, solar PV, small hydro, fuel cells, and biomass [4]. The non-traditional DG resources are environmentally friendly [[66], [67], [68]].

Other distributed energy resources (DER) that are also important are fuel cell systems and energy storage batteries employed for ancillary services in the grid-integrated system [[69], [70], [71]]. Energy storage applications and their usefulness are reported [[72], [73], [74], [75]].

The difference between traditional and non-traditional methods is that the traditional system has large generators with larger capacities than non-traditional methods with smaller capacities. Traditional methods can operate far from the end-user, whereas non-traditional methods are usually located near or at end-user premises for efficient energy delivery [76,77].

There are various merits provided by non-traditional over traditional DG technologies, such as economic, environmental, and technical benefits [62,[78], [79], [80]]. The economic benefits include emission reduction, fuel cost savings, and transmission and distribution operating cost savings. Environmental benefits are greenhouse gas emission reduction and natural resource conservation, and technical benefits include voltage stability, minimizing power loss, reliability, grid reinforcement, improvement in power quality, and secure supply. Table 3 gives benefits derived from different non-traditional DG technologies [81,82]. However, due to several merits of non-tradition DG, some technical and economic drawbacks still exist in integrating DG in power distribution networks. Inadequate placement of DG and sizing can lead to voltage instability, high power losses, failure in power quality, and protection in power system networks [[83], [84], [85]].

**Table 3:** Benefits derived from different non-traditional DG over the traditional technologies [81,82].

Non-traditional DG technologies	Economic benefits		Environmental benefits		Technical benefits	
	Reduction in operating cost of transmission	Reduction in cost of fossil fuel	Greenhouse gas emission reduction	Reduction in noise pollution	Improvement in power security supply	Dipatchability
Micro-hydro turbine	Yes	Yes	Yes	No	Yes	No
Biomas	Yes	Yes	Yes	No	Yes	Yes
Wind turbine	Yes	Yes	Yes	No	Yes	No
Solar PV	Yes	Yes	Yes	Yes	Yes	No

#### 2.1.1 Traditional DG resources

##### 2.1.1.1 Microturbine generation energy (MTGE)

The basic MTGE contains a compressor permanent magnet generator, and the turbine is placed on a single shaft. The incoming air was first reduced and passed to the component of the heat exchanger to increase the temperature through hot exhaust gases (recuperator) [76]. The compressor was obtained by increasing the pressure to 3–4 times the atmosphere. After the air had been reduced and heated, fuel was added to the combustion chamber and burned. The turbine then expands the hot gases to turn the generator and compressor. A small gas turbine ranges from 0.5 KW to hundreds of KW capacity [86].

**Fig. 3:**
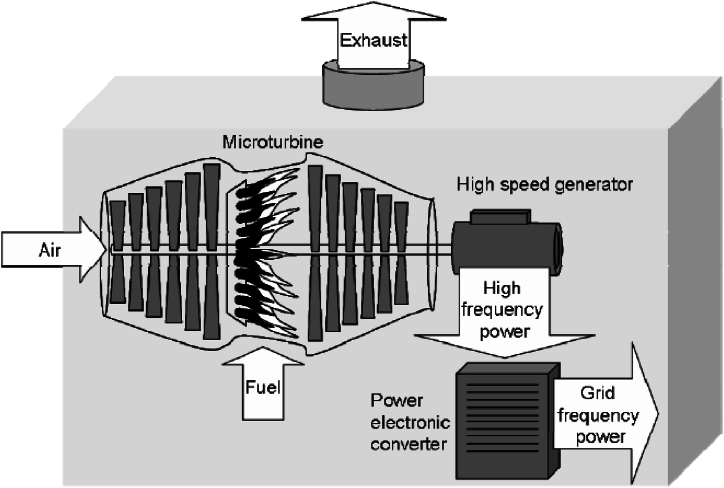
Microturbine generation energy [90].

The heat energy developed by microturbines has the advantage of combined heat and power application [87,88]. MTGE was used to create modular packages with a capacity above 1 MW. The arrangement was incorporated into the existing DN, but the machine will be synchronized [89]. A typical diagram of a microturbine generation energy is given in Fig. 3.

##### 2.1.1.2 Internal combustion engines (ICE)

These traditional-based DG resources use a reciprocating system called internal combustion engines. This type of power generation operates from 0.5 to 6500 KW. The efficiency is 0.4 on the smaller heating value. The ICE is driven by a piston and then connected to constant-speed alternating current (AC) generators. The reciprocating system is derived from natural gas, propane, fuel oil, gasoline, and water treatment plant digester gas [86]. This ICE has a low cost when compared to other types of DG, it is applicable in a cogeneration system, and it has a small environmental impact when it is derived from its source or fueled by natural gas resources [42,86,89,91,92].

Most internal combustion engines operate with a four-stroke cycle, similar to trucks and automobiles. The cycle includes power, compression, intake, and exhaust stroke. The diesel cycle and Otto cycle are variants of a four-stroke engine. Both are regarded as spark-ignited (s-i) and compression-ignition (c-i) systems. Fuel or gasoline can be easily ignited and used on spark-ignited engines, while fuel oil or diesel can operate diesel engines [86].

##### 2.1.1.3 External combustion engines

External combustion engines (ECE), e.g., steam cycle power plants, are called Stirling engines. At higher temperatures, sources like concentrated solar ECE can be operated. ECE benefits from cogeneration application, but emissions are generated when the fossil fuel is run, which can change the fuel price. It is environmentally friendly and silent in operation due to the energy that burns steadily and slowly. The capacity is up to 25 KW, but the efficiency is low, i.e.,< 0.3 [86].

#### 2.1.2 Non-traditional DG resources

##### 2.1.2.1 Wind turbine generation energy (WTGE)

**Fig. 4:**
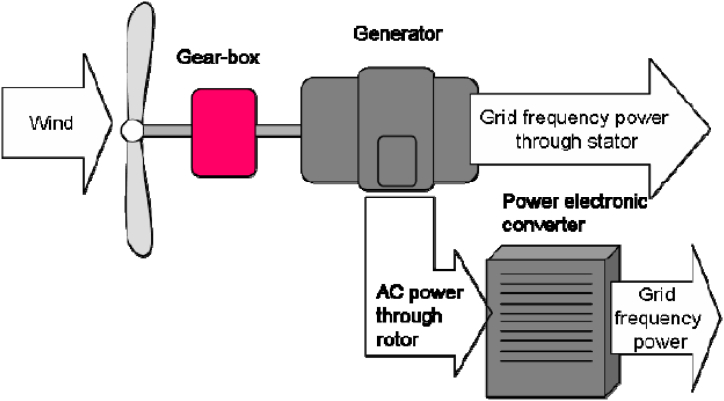
Wind turbine generation energy [90].

WTGE DG-based is non-traditional energy generation. The source of electric energy generation is wind [93]. Much work has been done on integrating distributed wind power across the globe. The problems and opportunities associated with on-grid distributing wind power in distributed networks were reported [94]. WTGE has intermittent characteristics that use energy storage systems (ESS) to store the energy when the wind blows, which may not coincide with peak periods [95]. Operation issues and technical benefits of integrating distributed renewable energy technologies with the existing electrical grid network are present [96]. Apart from the technical issues of WTGE, emphasis has been made on the power system, and the impact on transmission planning, power imbalance, operation cost, and power system dynamics has been reported [97]. Ibrahim et al. [98] identified emerging challenges that need attention, overview, and trends in power electronic converters utilized for wind power system production [98]. The challenges and the solution of the grid-integration WTGE system were discussed [98]. Fig. 4 illustrates a diagram of wind turbine energy generation.

##### 2.1.2.2 Biomass

Besides, energy generation is done through thermal, where steam is used to operate the turbine and the alternator is spun, distributing energy through biomass using waste heat, such as CHP [99]. Many research studies have been conducted on distributing energy through biomass. The challenges of biomass moisture that reduce the resource's calorific value and the combustion temperature were reported, leading to operating challenges [100]. This problem leads to drying biomass in the combustor before it is converted for electricity production. A multi-stage drying process was introduced to utilize waste heat and steam from the drying process and biomass plant. The method improved the biomass power system [100,101]. Another integration of biomass power system generation and gasification emphasizes distributing power system applications [102,103]. A hybrid system is an emerging trend in biomass technology; the power of the gas-biomass oxy-combustion hybrid system was considered for energy application, and its integrated aspect was reported [104].

##### 2.1.2.3 Fuel cell generation energy

**Fig. 5:**
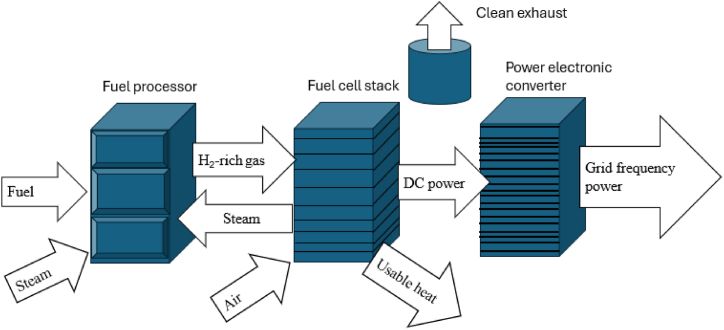
**Fuel cell generation energy** [114].

Among the energy resources that are distributed is a fuel cell that converts chemical energy to electrical energy as long as there is a continued provision of oxidant and fuel [66,105]. Heat energy generation forms water in the energy conversion process, a by-product [106]. Research has proved that fuel cells are applicable in DG and CHP [[107], [108], [109]]. The technology is helpful for both on-grid and off-grid applications. Difficulty of fuel cell have been reported in [[110], [111]] due to the technology with limited load-following capacity. Therefore, a hybrid configuration solves limited load-following capacities such as fuel cells, batteries, and microturbines. However, power electronics, effective control mechanisms, and grid synchronization must operate and integrate with the existing distribution grid [112,113]. An illustration of the fuel cell generation energy is given in Fig. 5.

##### 2.1.2.4 Storage Battery

The storage battery is an alternative way of storing energy for an alternative use when grid energy has been taken off. It is used for essential services in grid integration applications like power quality, regulation of frequency and voltage, energy management support, and bridging power. The support of voltage regulation of the battery system is a viable approach for voltage stability when DG units are deployed in distributed networks [76]. Kumar et al. reported a new optimization technique for RE storage systems for power quality analysis [115]. The authors suggested trading and control in the DC microgrid of day-ahead power management. The multiobjective optimization dispatch (MOOD) problem was used to reduce the operation cost and the cost associated with power loss. The method was reported to be superior to other techniques in the literature [115].

##### 2.1.2.5 Solar PV energy

In a DG, solar energy is derived from sun sources and fueled by solar energy resources [116,117]. However, DG's solar energy is grouped into solar PV and thermal systems [118,119]. In an off-grid system, battery storage is required to store the energy generated by solar PV during the day. Still, battery storage was not needed for application in specific grid-connected systems. An inverter is required in both off-grid and on-grid systems since it converts the direct current (DC) generated by solar PV to AC to be used by the household appliance and the grid [67].

Much work has been done to advance solar PV systems. For example, Alam et al. [120] reported the evening peak as a period to manage the availability of stored energy systems. The reduction of rooftop solar PV was also presented and recognized. A high proportion of rooftop DG solar PV into low voltage (LV) DN led to backward power flow and increased voltage profile. A way to study the problem is when solar generation goes beyond load demand with high solar radiation. Also, energy distribution storage (EDS) system with solar PV is integrated into LV networks to store the excess energy and used when there is no sun to charge the battery, e.g., during evening periods. The discharge and charge control system for battery energy (BE) is discussed based on the depth of discharge, charging state, and period of charging times.

An evaluation of power quality on grid-solar PV integrated power generation was presented [121]. A distribution network has a passive character with power flow. The conventional power flows from DN in a single direction to the substation, the load center, and the feeder. Integrating solar PV generators with DN alters the behavior, design, and grid characteristics, which may affect the power quality of the electric power system.

**Fig. 6:**
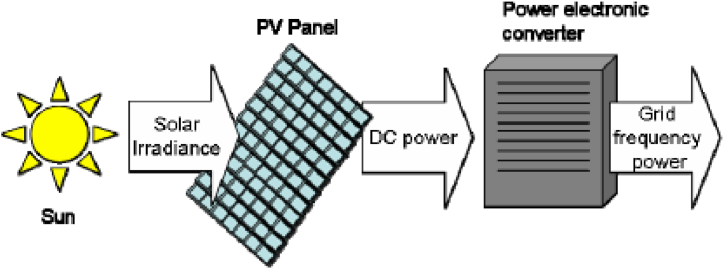
**Solar PV generation energy** [124]**.**

Furthermore, Obi and Bass reported the application of solar tracking, maximum power tracking charge controller, and transformerless DC-AC inverter/converter to get efficient energy from solar PV systems with lower impact on the existing distribution grid. Also, the development of the on-grid solar PV systems, the challenge of higher penetration of DGs as potential pressure on the current DN, and the techniques to handle the issues associated with the increased deployment of on-grid solar PV systems were reported [122]. A comprehensive review of PV-DG allocation in power systems and solar energy resource potential assessment was reported [123]. It was reported that PV-DG installation and sizing was a great way to use clean energy's merit [123]. Fig. 6 illustrates the Solar PV generation energy diagram.

## 3 Impact of DG on voltage stability

**Fig. 7:**
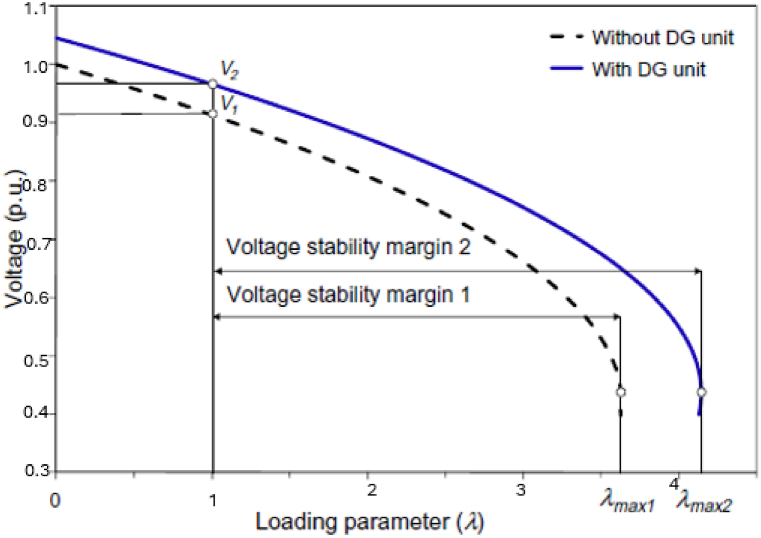
Impact of DG on maximum load-ability and voltage stability margin [126].

Various techniques have been used to describe voltage stability analysis, including dynamic and static methods. Static methods can be described using the relationship between the power (P) at the receiving end and voltage (V) at the receiving end in a particular node in a system, which is called the noise curve or P-V curve, as shown in Fig. 7. A continuous power flow equation was applied to obtain a P-V curve [125]. A saddle-node bifurcation factor (critical point) $\lambda_{\max}$ in the P-V curve is used to represent maximum loading in the system and the point is the singularity of jacobian power flow equations.

The stability margin is the distance between the initial operating point and the critical point, and its unit is the megawatt (MW). The impact of DG units in the distribution system can either raise or reduce the voltage stability margin depending on the power factor's value, unity, lag, or lead.

Nowadays, most DGs installed are operated at a unity power factor (PF) to avoid any unnecessary inference that may occur with the connected voltage regulator [127,128]. It is assumed that every DG unit is operated at a unity PF. However, some utilities allow their DG unit to work at a constant PF, usually between 0.95 leading and 0.95 lagging.

Fig. 7 represents the loading parameter (λ) in the x-axis, which varies from 0 no load to the maximum loading point $\lambda_{\max}$. With the injection of real power into the DG unit, the voltage operation point increases from $V_{1}$ and $V_{2}$ and maximum load-ability increases as well $\lambda_{\max\;1}$ and $\lambda_{\max\;2}$ as shown below. Fig. 7 shows the benefit of the DG unit on maximum load-ability and voltage stability margin.

$$Q_{i}=\lambda \;Q_{i}$$

Over the last decade, DGs have been considered to supply real power energy. The system operates at a high loading rate with higher DG penetration, so the poor voltage profile has been challenging for power utility companies. Reactive support compensation must be placed to maintain voltage limits at a permissible rate [129]. Hu et al. reported the reactive power probability distributed energy resources for voltage stability in DN. Relative available transmission capacity index (RATCI) is proposed to evaluate the voltage stability by integrating DER. The result shows that with multi-type and location of DER in DN, voltage stability was improved [130]. Allocation of DG in the system affects the reactive power management plan [131]. When asynchronous induction and wind generators are used, the generator must support a reactive power from which the system is connected. We have many methods of reactive compensation, such as shunt capacitors, synchronous generators, and end-user reactive power compensation with reactive power consumption equipment. Solar and wind energy (non-conventional) power generation have been used to provide a reliable source of energy voltage profile and reactive power support to the system.

Recently, there has been growth in technology from DG that controls both reactive and active power. Wind generation is now used as a double-fed induction generator. The solar PV used a unique self-commutated line inverter to absorb and supply reactive power at separate loading. The reactive power capacity of wind and solar power plants can be improved using a static synchronous compensator (STATCOMS), static var compensator (SVC), and various forms of reactive power support to the system. Inverter-based reactive support is costly compared to other synchronous machines of the exact sizes [132,133].

The importance of reactive power in the presence of DG, which improves the voltage profile, was reported [131,132]. Moreover, the load was reduced in the distribution system with the presence of DG, and the issues of rising voltage may occur. It is necessary to have voltage regulation in the system. The curtailment of energy from DG is not a proper way to result in revenue loss. It was reported that DG is an active energy source [[134], [135], [136]]. Furthermore, DG has been used for active and reactive energy sources [[137], [138], [139], [140]]. Different DG types were reported [141,142].

## 4 Optimal planning of DG in the distribution system to minimize loss

**Fig. 8:**
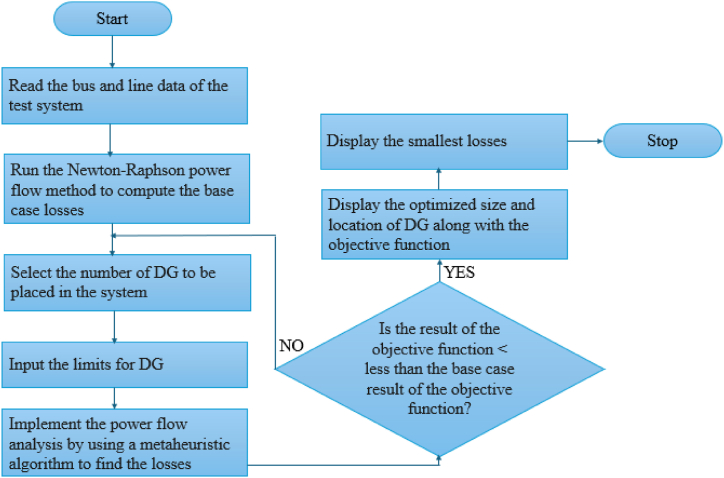
Flowchart of loss minimization with DG.

The optimum sitting and sizing of DG are very important in the power system to reduce loss in the design and improve the voltage profile in the distributing system (DS). For this to be an archive, many researchers have applied many approaches, such as intelligent, conventional, and hybrid methods, to minimize energy and power loss. Fig. 8 illustrates the flowchart to minimize losses (i.e., energy and power loss) using the meta-heuristic algorithm.

### 4.1 Energy loss reduction for DG planning in a distribution system

Hung et al. [143] give three (3) analytical methods on Elgard's branch power loss formula and branch current to get optimum PF and proper placement of DG units. The energy loss was reduced when considering varying load times and continuous types of multiple DGs. The method takes less time when compared with the exhaust load flow (ELF) method; the total energy loss per annual was obtained, as shown in Equation (1). Also, one dispatchable/non-dispatchable renewable energy based on DG was observed.

(1) $$E_{loss}=365\sum_{t=1}^{24}P_{Loss}^{t}\Delta t$$

where $P_{Loss}^{t}$ is the actual power loss and Δt, and t is the duration of time, 24 is the total number of hours per day, 365 is the number of days per year, and the total of hours per year is 8760 h (24∗365).

Dispatchable DG units are more influential in voltage profile improvement and energy loss reduction than non-dispatchable DG units [144,145]. The analytical expression was modified, giving a better optimum allocation of dispatchable and non-dispatchable DG units. Furthermore, analytical expression was used for optimum sizing, and allocating three types of DG units was reported to reduce loss [146,147]. The formula is given in Equation (2), which calculates the hourly load for 96 h for one year. Artificial neural networks were presented to reduce yearly energy loss; solar PV DG was used in low-voltage distribution networks [148]. Also, the usefulness of PSO has been reported in planning models appropriate for dispatchable and non-dispatchable DG allocation in distribution system networks [149]. An hourly planning problem was formulated to locate the optimum production profile, PF, and DG unit piercing. The generation load profile is assumed to be a day (24 h) [149]. A multi-objective pareto-based velocity butterfly optimization algorithm (MOVBOA) was reported to minimize energy loss, yearly economic cost, and voltage deviation. MOVBOA gives the best results compared to multi-objective (MOBOA) and NSGA-II [150]. The formulae used to obtain the energy loss per annum for only a generation load profile of 1 h is given in Equation (3).

(2) $$E_{loss}=91.25\sum_{t=1}^{96}P_{Loss}^{t}\Delta t$$

(3) $$E_{loss}=\sum_{k=1}^{Nh}P_{k}^{loss}\left(X\right)$$

where $P_{k}^{loss}$ is the real power loss equivalent to Nh hour, and X is the vector variable, 96 is the hourly load curve of a day for each season. Each year has four seasons. Therefore, the load curve for four seasons is 24-h days (4∗ 24 = 96 h). 91.25 is the repeated load curve for 96 h for one year (i.e., 96∗91.25 = 8760) [144].

Furthermore, the Krill herd optimization (OKH) technique was reported to find the size of the renewable and optimum site of DG to reduce yearly loss in energy, and the geographical and environmental constraints are nullified [151,152]. A mixed-integer nonlinear programming (MINLP) method was used to formulate the planning and optimal allocation of various renewable DG units such as biomass, solar, and wind energy [[153], [154], [155]]. A generation load model was reported, including the possible operation of renewable DG units with various demand possibilities to minimize yearly energy losses [153,154]. The relationship between load and wind energy is not considered, and the study period was divided into many segments and treated separately [156]. The objective function for yearly energy loss minimization is given in Equation (4).

(4) $$Minimize\;cost=\sum_{g=1}^{N}P_{lossg}\times P\left(C_{g}\right)\times J$$

where $P\left(C_{g}\right)$ is the probability of load combination and wind-based DG output, $P_{lossg}$ is the power losses in the distribution system, and N is the number of discrete states, J is taken between 90 and 8760 were reported [153,154].

Khatod et al. [157] give renewable energy generation adapted to probability techniques. Two operations strategies were adopted for the stability of voltage networks. The first one is off the wind turbine generator, and the second is the clipping wind turbine generator output until wind energy is dispatched to the system percentage loads. The objective function is given in Equation (5).

(5) $${minimize\;F}_{C}={AE}_{loss}^{E}$$

where $F_{C}$ is the objective function to minimize, ${AE}_{loss}^{E}$ is the active energy loss over the study period.

### 4.2 Power loss minimization in distributing system using DG

At each stage of the electrical power system is the presence of losses, i.e., generation, transmission, and distribution, caused by the aging of equipment, unbalanced load, harmonic distortion, tripping of lines, etc. The power is kept at a high voltage to reduce the transmission and distribution center losses. However, this does not eliminate the loss at the consumer and distribution, and it's essential to place DG near the load center (i.e., the consumer end) to reduce the losses and improve the voltage profile [81,158].

The loss reduction formulation was based on three methods: current loss, branch power loss, and exact loss [[159], [160], [161], [162], [163], [164], [165], [166], [167], [168]]. Several researchers have used the methods mentioned above to site and size DG to minimize losses and improve system functioning. The formulation for current loss has been reported [143,169], and their optimized planning tasks were reported [[170], [171], [172], [173], [174], [175], [176], [177]] according to Equation (6). The objective of power loss has been formulated by Refs. [143,178], according to Equation (7), exact loss formulae were developed by Elgerd I.O [179], which is also known as loss formulae [180], based on Equation (8). The previous studies on loss minimization based on Equation (8) are [142,[181], [182], [183], [184]].

(6) $$P_{Loss}=\sum_{i=1}^{nb}{\left|I_{bi}\right|}^{2}R_{bi}$$

(7) $$P_{Loss}=\sum_{i=1}^{nb}\left[\frac{P_{bi}^{2}+Q_{bi}^{2}}{{\left|V_{i}\right|}^{2}}\right]R_{bi}$$

(8) $$P_{Loss}=\sum_{i=1}^{Nb}\sum_{j=1}^{Nb}\left[\propto_{ij}\left(P_{i}P_{j}+Q_{i}Q_{j}\right)+\beta_{ij}(Q_{i}P_{j}-P_{i}Q_{j}\right]$$

here

$n_{b}$ = total number of branches.

$V_{i}$ = voltage magnitude of i bus.

$P_{Loss}$ = Total power losses on the distribution system.

$R_{bi}$ = branch resistance of i branch of the distribution system.

$X_{bi}$ = branch reactance of i branch of the distribution system.

$I_{bi}$ = current magnitude of i branch.

$\propto_{ij}$, $\beta_{ij}$ = loss coefficients.

$Q_{bi}{,P}_{bi}$ = reactive and active power flow at the i branch of the system.

$Q_{i}{,P}_{i}$ = net reactive and active power flow at i bus of the distribution system, respectively.

$Q_{j}{,P}_{j}$ = net reactive and active power flow at j bus of the distribution system.

PSO algorithm was used to investigate DG's best sizing and placement for power loss reduction and voltage profile improvement [185]. Adaptive Shuffled Frogs Leaping Algorithm (ASFLA) was implemented for the location and sizing of DG and network reconfiguration to reduce power loss and voltage stability improvement; it was reported that the ASFLA was more effective than the other algorithms like SFLA, WA, and ACSA [186]. A filter Kalman algorithm investigates the optimal sizing of DG to diminish the power loss and improve voltage profile [187,188]. An analytical method was reported for the sizing and siting of DG to reduce power loss in the radial distribution system. Multiple and single DG have been considered [189]. A concentration load bus concept was used to find the location of each candidate. The drawback was overcome, and more planning practical solutions took to 10 KW DG steps size [159]. A sensitivities-based approach to power loss optimum locator index (OLI) minimizes the computation time. The higher value of OLI indicates the proper place for DG placement [159]. The formulation of OLI is given in Equation (9).

(9) $${OLI=w}_{1}\frac{P_{M}}{P_{M,\max}}-w_{2}\frac{P_{loss}^{sensitivity}}{\left|P_{loss}^{sensitivity}\right|\min}$$

where

$w_{1}\;and\;w_{2}=$ are the weighting factors.

$P_{M,\max}=$ maximum power margin.

$P_{M}=$ power margin.

$P_{loss}^{sensitivity}=$ power loss sensitivity and minimum power loss sensitivity is given in terms of $\left|P_{loss}^{sensitivity}\right|\min$.

Mekhamer et al. [190] reported the limited types of DG and analytical expression to find the optimum placement and power factor (PF) of different DG units, which require less time in computation. The optimal ${PF}_{DG}$ was found in mutual agreement with the ${PF}_{load}$ [190,191]. [184]. In the radial distribution system, an investigation was carried out to determine the optimal siting and sizing of multiple DGs. PSO algorithm was reported to diminish the power loss and improve the bus/node voltage, and DG was considered one after the other [192,193]. A cuckoo search algorithm (CSA) was reported for the sizing and placement of DG and capacitor banks to minimize active and reactive power loss. DG and capacitor bank are placed on DN simultaneously, and the result reveals that CSA effectively reduces active and reactive power loss [194]. A modified flower pollination algorithm (MFPA) and fitness-based balance (FDB) were used to determine DG's size and location to minimize power loss. The authors considered various load types and convergence times together with minimizing power loss. The results reveal that MFPA was superior to all other methods in the literature [195]. A systematic review of optimal planning and deployment of DG and energy storage systems (ESS) in DN was reported, along with the advantages and disadvantages of DG and ESS. It was reported that the placement of DG can elevate the problem of voltage instability, lower the overload of feeders, and improve the power grid reliability [196,197]. An improved PSO (IPSO) was presented to size and allocated DG to reduce the power loss and improve the voltage profile considering the different types of DG (type-1, type-2, and type-3 DG) [198]. A selective PSO (SPSO) was presented for optimum reconfiguration and allocation of DG and capacitor bank in the distribution system. The power voltage sensitivity constant (PVSC) was formulated to determine the bus for DG placement. The authors considered three types of load conditions (i.e., light, normal, and heavy load). The reduction percentage for real power was improved by 99.341 %, 97.289 %, and 95.389 % for each load condition [199,200]. Hybrid PSO and pathfinder algorithm (PFA) (HPSO-PFA) were proposed to minimize power loss in electrical power systems. The results of the proposed HPSO-PFA give the best results out of all the methods compared with such PSO, GWO, MFO, SARGA, PFA, ALO, etc. [201]. An adaptive modified whale optimization algorithm (MWOA) was reported for optimum DG allocation (ODGA) and network reconfiguration (NR) power loss reduction. NR and ODGA improved the voltage stability profile considering probabilistic load and DGs by varying power factors (PF). The result was compared with CSA, CGA, and runner root algorithm (RRA); the proposed MWOA outperformed them [202]. Archimedes optimization algorithm (AOA) was reported to reduce greenhouse emissions, and a loss sensitivity factor was used to find the place for solar PV. The result was compared to PSO, ALO, CSA, TLBO, etc, as the losses were reduced, and the voltage profile was improved when solar PV was placed [31]. PSO and DE were used to identify DG's best sizing and location using the DG suitability index (DGSI) [203]. The power loss reduction was made using PSO and DE to allocate sixteen DG on the 33 bus system. The authors reported an improved voltage profile after DG installation [203]. Hussain and Roy used a modified artificial bee colony (MABC) for OADG in a minor-scale distribution system, which gave a good solution and less computation time. The voltage profile improved, and losses were reduced in the distributing system when considering DG inequality constraints, e.g., DG boundary, thermal limits, and bus voltage limits [171,204]. A multi-objective optimization was reported to allocate DG to minimize voltage deviation, power losses, and annual energy loss [79]. Cook bird optimization (CBO) was reported for the location and sizing of DGs to minimize power loss and voltage deviation. CBO was compared with other methods, and CBO gave satisfactory results [205]. A techno-economic and optimal allocation of DG to minimize power loss using WOA has been reported. WOA gave a superior result compared to the other methods [206]. Polar bear optimization (PBO) was reported for the location and sizing of capacitors in RDN. Power loss reduction has been considered as the objective function [207]. In another study, GA and ACA were reported for DG location and sizing to minimize power losses [208]. In addition, reactive power compensation has been reported for enhanced voltage stability and security using DG for optimum location and sizing [140]. Pareto optimality and technique for order of preference by similarity to ideal solution (TOPSIS) approach has been reported for multiobjective techno-economic accommodation of DGs in DN [209]. A harmonic and frequency power loss was reduced using the OADG based on a combined tabu search (TS) and GA. The proposed hybrid TS-GA gives a better result than the one obtained by GA [4,210]. A Thumb rule was presented for DG capacity and quantity identification. The objective function (f) is given in Equation (10).

(10) $$f=\sum_{j=1}^{F}\left[\sum_{i=1}^{Bj}P_{ij}+\gamma \left(\sum_{k=1}^{Lj}P_{kj}-C_{j}\right)+P_{hj}\right]$$

here F = is the overall radial feeders in the distribution system.

γ = penalty weight.

$P_{ij}=$ node inject power at bus i at the j feeder.

$L_{j}=$ total number of load buses at the j feeder.

$P_{hj}=$ power losses of the harmonic at the j feeder.

$P_{kj}=$ load power of bus k at the j feeder.

$B_{j}=$ is the overall number of busses at the j feeder.

$C_{j}=$ overall injected power of dispersed generations at the j feeder [210].

The analytical formula for the actual loss was presented for optimum DG efficiency and rating [180]. The load flow was performed to test its effectiveness; two cases were considered: without DG placement and with DG placement. The Impedance matrix was denoted by (Zbus), and the bus admittance matrix, which was represented by (Ybus), is required for OADG, and voltage constraints were ignored [211]. A sensitivity analysis for OADG was carried out by Ramesh et al. [170], for a fixed load impedance model to reduce active power loss and study bus voltage at 11 kV distribution feeder in Indian Electricity Board TNEB and IEEE 37 bus system [133]. The simultaneous solution with the optimal siting and sizing problem (SSOSSP) method was done using a harmony search algorithm with the differential operator (HSDO) to find the exact size and location of every DG unit in an individual/single go. The loss sensitivity factor (LSM) and voltage sensitivity factor (VSM) were reported. The results show that SSOSSP gives quality solution in power loss reduction [212]. TLBO and modified TLBO algorithm [213,214] was used to study the optimum allocation of DG units to minimize power loss. TLBO needs fewer parameters, such as the maximum number of iterations and cross-over rate, population size, and a global solution. The implementation is simple and requires small computation memory compared to other search methods [156]. Moreover, the answer may violate the constraint operation of DG, like bus voltage and current branch limits, because they were not considered in mathematical modeling [215].

Implementation of the PSO algorithm was reported [142,181] to allocate proper rating and placement of multiple types of DGs and optimum ${PF}_{DG}$ it was also located when taken assumptions on a fixed power load model. The results were compared with the GA and artificial bee colony (ABC) algorithm as given [208,216] for 69 bus systems. There are minor changes in DGs compared to both meta-heuristic algorithms, but there are few changes in the percentage of active power loss against the ABC method. A sensitivity analysis was applied [172], considering the characteristics of the constant current load. The buses 35–69 are considered for the proper location of a DG in 69 long bus feeder [172]. Expression for power loss reduction (PLR) depends on the current loss formula [173]. The PSO algorithm was used as an enhancement tool for the efficiency of PLR and voltage profile improvement of a typical Nigerian grid.Furthermore, the operational constraints of DG are not mentioned. The advantage of EP and PSO (REPSO) is that they obtained solutions to the same objective within a limited period [173]. The exact value of PLR was obtained after the placements of two DG [174,192]. The authors [173,177] reported the objective function in Equation (11). When DG is present, J is one (1); otherwise, it is zero.

(11) $${PLR}_{i}=\sum_{i=1}^{nb}\left({2JI}_{bi}I_{DG}+{JI}_{DG}^{2}\right)R_{bi\;}$$

where $I_{DG\;}$ = distributed generation.

The bat algorithm (BA) and gravitational search algorithm (GSA) were presented for sizing and location of DG to reduce power loss by incorporating solar PV [217]. A forward/backward sweep was used initially to analyze the power loss [217]. BA was used to reduce the power loss with the changes in all DG nodes [218]. The limit values for bus voltage, both upper and lower limits, are 1.1 and 0.9, respectively. Jamian et al. present that the hybrid REPSO method was more potent than conventional PSO, inertia PSO, and iteration weight PSO. Moreover, the multiple allocations of DG units were later pre-decided [175]. In the analytical approach, the first thing to be done is to locate a sequence of buses where DG units are allocated [176]. The output of DG units was obtained at the identified point by optimizing the loss-saving equation. Also, the method is not suitable for meshed and unbalanced distribution networks. The PSO solution for DG unit sitting sizing has been given in the form of parameter selection. At the peak demand, 50 % of the light load was considered, and 150 % of the heavy load was considered at the peak demand. Furthermore, DG was reported to supply only the actual power to the grid. Kansal et al. [219,220] reported that using the analytical method (AM) alone is inappropriate for the optimum placement of many DG units. It comes up with a hybrid approach. The method used AM to calculate the output of DG units at every node; therefore, the PSO algorithm was used to address DG units. The artificial neural network-based optimum solution was reported to answer OADG challenges with load changes, VAR of many DG, and their piercing/penetration. The training algorithm used is resilient backpropagation, which is more potent than the steepest descent algorithm [221]. There is an improvement in the performance of bacterial foraging optimization (BFO), and it is called modified BFO (MBFO) [163]. The MBFO was used for DG allocation. The result was adequate and had convergence speed when compared with BFO. Karimyan et al. [182] reported a new approach for long-term optimization schedules for variable planning in the radial distribution system by improving the voltage profile and reducing active power losses. The DG type and linear step change load are considered. The nature of the problem and penalty function method (PFM) were added to the new PSO algorithm to meet the inequality and equality constraints [182,222,223]; the technique is easy to apply and simple in the load level. The AM techniques were employed to validate the proposed method. Furthermore, the PSO method has more computation time than AM.

The efficiency of analytical methods was reported by Ref. [163], which can be used in various kinds of many-type DG units and different types of optimum mix with many generation capabilities. The approach used optimal power flow (OPF) methods to handle the overall constraints [163]. The loss sensitivity factor was used to obtain the optimal bus. Also, DG size was optimized based on the harmony search algorithm (HAS), and intelligent water drop (IWD) was used to reduce power losses [164,224]. Moreover, a single DG unit was placed [164], and the placements of many DG units in the distribution system were reported [224]. GA was used to identify the most appropriate generator type for DG and obtain the siting and sizing of the DG unit to minimize losses. Asynchronous, synchronous, and induction generators based on DG technology are used as various parts of the optimization problem. The method was tested on the IEEE 14 bus system [165]. The modified firefly algorithm (MFFA) was reported by Othman et al. [166] to find the optimal sizing and sitting of DG units without fail the practical constraints of the distributing system. A Modified Aquila Optimizer (MAO) was presented to reduce the power loss and voltage deviation considering multi-objective function for optimum DG placement. The method was reported to reduce the objective function effectively [225].

## 5 Voltage stability (VS) improvement-based DG for optimal planning in the distribution systems

VS improvement can be achieved if proper sizing and optimal placement of DG are done, and it will prevent the system network from any harmful occurrence. Therefore, many researchers have proposed multiple innovative solutions in voltage assessment techniques, and methods of solving voltage instability issues have been formulated.

### 5.1 Improvement of voltage stability based on DG planning

DG should be placed on the weakest bus in the system that is liable to voltage collapse, which many researchers have worked on.

Ayalew et al. reported the integration of renewable energy for network expansion and voltage profile improvement; it was reported that the Addis North feeder-1 (ANF) distribution network was upgraded when RE was incorporated into the system, and the voltage profile was improved [226]. An improved modal analysis technique (IMAT) was reported to identify the weakest bus liable to voltage collapse in PS [227]. IMAT was based on the Jacobian submatrix to analyze voltage stability. The method was compared with conventional modal analysis and the existing voltage stability indices. The IMAT gives a better performance than the other methods [227]. The line stability index and fast voltage stability index were reported to identify the weakest bus in PS and avoid voltage collapse using IEEE 9 and 14 buses [228]. The authors used enhanced PSO and other PSO variants like RPSO, TVAC-PSO, and PSO based on success rate (PSO-SR) to minimize the power loss. The obtained result proved that EPSO gives better results [228]. Hybrid PSO was reported for multiple DG considering current carrying capacity, DG penetration, and voltage magnitude constraints to maximize network loadability [229]. The IEEE 33 bus system was used to test the performance of HPSO. The result reveals that with multiple DG, the loadability of the system is enhanced. The authors concluded that with DG placement, the distribution company would benefit from maximization in loadability. In addition, another author [165] reported the impact of GA techniques for sizing and allocating DG in the IEEE 14 bus system. Three types of DG were applied to the system to minimize power loss. The result of GA was reported to be suitable for sizing and placement. It was further reported that multiple DG-reduced power losses [165]. The power loss index was used for the selection of the bus for placement of DG, and the WOA optimization algorithm was used for sizing. The results reveal that with type III DG, the best results were obtained, and WOA gives the best result compared to others [230]. Composite load modeling using power stability index, VSI, and line stability index was reported using PSO, improving PSO (IPSO) and TVAC-PSO for multiple DG allocation to minimize power loss and stability of voltage. The result shows that TVAC-PSO performs better than traditional PSO and IPSO [231]. Reactive power compensation was presented to improve the voltage profile and reduce power loss by integrating renewable distributed generation (RDG) into the conventional power system to enhance the voltage profile [140]. The result obtained was compared to PSO, ALOA, MLPSO, BPSO, and GA; the proposed method gives more satisfactory results than others [140]. The delta-star stator winding switching of the induction generator was presented to lower the sink of reactive power coming from the grid. However, there was an improvement in the node voltage [232]. Raja et al. improved the method, which integrated the permanent parallel capacitor at every line of three-phase winding. It gives better performance in the squirrel cage induction generator (SCIG). The load flow result was used to determine VCI, which gives helpful information near the CPF method with little effort in computation. Also, a bus with a smaller value of VCI will need a SCIG-based DG to adjust the voltage at the bus. Various ways of configuring SCIG were reported [233]. When adjustment is not involved, the VCI approach can be practiced in a complex system [233]. The equation for the loadability factor (LF) at the receiving end was presented [234], and the equation was revised [235]. The Equation measures the LF multiplied by load demand (i.e., loadability limit) at every bus in the system before voltage collapse occurs. Equation (12) given below was used [235].

(12) $${LF}_{i+1}=V_{i}^{2}\left[\frac{-\left(P_{i}R_{bi}+Q_{i}X_{bi}\right)+\sqrt{\left(R_{bi}^{2}+X_{bi}^{2}\right)\left(P_{i}^{2}{+Q^{2}}_{i}\right)}}{2{\left(P_{i}X_{bi}-Q_{i}R_{bi}\right)}^{2}}\right]$$

CPF method was used to determine the voltage security limit [236], the sensitive mode and the surrounding nodes were selected based on the success of the MA, and the given parameter of the load was carried out and compared with another index, proved as adequate for DG placement. When insufficient, the modified equivalent reactive compensation (MERC) methods present a list of nodes for VAR compensation and study the changes in DG units on voltage profile on active and reactive power losses. However, the method does not give an optimal solution [138].

The voltage profile sensitivity method was used to study each node for wind and solar energy-based DG placement [237]. The MINLP was used to formulate planning variables when considering the probabilistic type of load and solar energy DG placement. Due to problem formulation, only the main node feeder was considered to accommodate DG [237]. A method was presented to select the appropriate DG type and the best optimum location to improve voltage stability [238]. The MA and CPF methods were tested on IEEE 33 and 136 bus distribution systems. Ntombela et al. reported the system reconfiguration, sizing of DG, and placement for power loss reduction and voltage profile using HGAIPSO [239]. The method was compared with algorithms like GA and PSO; the HGAIPSO gave a promising result in the DN reconfiguration problem [239].

Improved pathfinder algorithm (IPFA) based inertia weight was reported for power loss reduction and voltage profile improvement. The results reveal that IPSO gives superior results to other methods in the literature, such as DE, PSO, MFO, GSA, GWO, etc [9]. A decision tree (DT) approach was used for multiple PVDG placement using some indices in unbalanced DN. The indices used are the branch risk index (RI) and power loss (PL). RI and PL indices are used to determine the placement of PVDG. The result was compared with ALO, SKHA, CSOS, SDE, etc., and the results of DT reveal that the method is adequate for voltage enhancement and power loss reduction [240]. An improved equilibrium optimization algorithm (IEOA) combined with a recycling strategy for optimum multiple DG placement was presented to improve the voltage profile [241]. The method was tested on standard 23 benchmark function, 33, 69, and 137 test systems. The method was reported to improve the voltage profile effectively and, as a result, was compared to HAS, EOA, FWA, and ISCA [241].

The issues of short-term measured and underground voltage-facing power systems caused the allocation of DG to the Abdul Rehman Baba grid station in Pakistan. Electrical Transient Analyzer Program (ETAP) was used to model the system to improve the voltage profile. The result reveals that the voltage profile was improved when DG was inserted into the system, and the overall efficiency was enhanced [242]. Steady-state VSI was reported to determine the weak node that can cause instability before placement of DG. Newton Raphson method was used to calculate the load and determine the system's steady state. The result shows that the system's stability should be considered before optimizing system distribution losses [243]. Mayfly Algorithm (MA) optimization method for placement of DG to improve the voltage profile. The IEEE 69 bus system was used to test the approach's performance, and three types of DG were incorporated into the system. The result reveals a 68.09 % reduction, significantly improving the voltage profile [244]. Optimal placement of DG using GA was reported to enhance the voltage profile and maximize loadability. The DG unit containing reactive power was used to raise a test feeder's voltage stability load-ability limit, and the security grid was improved under a single N-1 contingency. The result reveals that GA is suitable for maximizing voltage stability margin and enhancing voltage stability [245].

Steady-state security of the distribution network of DG based on the optimization planning technique was reported [246]. Mohammad Aryanfar presented a method of sizing and locating dispatchable DG at various levels to enhance the voltage profile for better system operation [247]. In Ref. [226], the DG placement was presented for optimal dissemination of actual power loss and increased maximum load-ability limit (VLL). Maximum demand is satisfied by the grid supply when the bus voltage at every node abides by the node voltage limits defined as VLL. Optimal planning was carried out at a constant load [246]. Also, the type of DG used determined the effects on the voltage system security have been reported [248]. The induction-type wind-based DG unit is to be placed in the most substantial region, which has the higher values of $Q_{loadability}$ The buses with lower index values should be supported with a solar energy DGs placement for better performance of VSM. However, a capacitor was put together with wind-based DGs [248]. The $Q_{loadability}$ index is given in Equation (13).

(13) $$Q_{loadability}=\frac{Q_{margin-new}-Q_{margin-base}}{Q_{margin-base}}\times 100$$

where

$Q_{margin-new\;}$ is the reactive power margin with DG.

$Q_{margin-base}$ is the reactive power margin without DG.

## 6 Multi-objective (MO) optimization for optimal DG planning

Multi-objective optimization gives a better approach to DG planning by implementing the best solution out of many single-objective functions. This section only considers voltage stability improvement and loss minimization as a single objective function. The weight objective function (WOF) combines the objective function mentioned above and the improvement of the voltage profile objective with weighting factors to form a single objective function called WOF. When the objective function is optimized separately or by other methods, the formulation of the problem is considered MO [4].

Pandey and Awasth reported that the harmony search (HS)-based hybrid genetic algorithm integrated adaptive particle swarm optimization (GA-APSO) method was presented to solve multiple-objective function (MOF). The target is to reduce the total cost generation index (CTI), actual power loss (APL), voltage deviation index (VDI), reactive power loss (RPL), and load balancing index (LBI) in IEEE 33 and 69 bus systems [249]. The formulation is given in Equation (14).

(14) $$MOF=minimize\left[\left(wf_{1}\times CTI+wf_{2}\times API+wf_{3}\times RPI+wf_{4}\times VDI+wf_{5}\times LBI+wf_{6}\times SBI\right)\right]$$

where $wf_{1}+wf_{2}+wf_{3}+wf_{4}+wf_{5}+wf_{6}=1$ and is the indices weight factors [249].

Non-Dominated Sorting Genetic Algorithm II (NSGAII) was reported to solve MOF to minimize power loss, operation, and maintenance costs. The NSGAII was reported to be adequate in handling the MOF [250]. The formulation of simple GA to raise the performance of OADGs in the Khoda Bande Loo test feeder distribution was presented [251]. It was reported that power loss was reduced and the voltage profile improved. The formulation for multiple-objective function (MOF) is given in Equation (15) [251].

(15) $$F=K_{1}\left\{\mathrm{Max}\lceil 0,\frac{1}{Nb}\sum_{i=1}^{Nb}\left({V\%}_{i}^{DG}-{V\%}_{i}\right)\rceil \right\}+K_{2}\left\{\mathrm{Max}\lceil 0,\sum_{j=1}^{nb}\left(P_{jloss}-P_{jloss}^{DG}\right)\rceil \right\}+K_{3}\left\{\mathrm{Max}\lceil 0,\sum_{j=1}^{nb}\left(Q_{jloss}-Q_{jloss}^{DG}\right)\rceil \right\}$$

where

${V\%}_{i}^{DG}$ = percentage of voltage in i bus with DG.

${V\%}_{i}$ = percentage of voltage in i bus without DG.

${Q_{jloss},P}_{jloss}=$ reactive and active power losses at j branch without DG.

$Q_{jloss}^{DG},P_{jloss}^{DG}$ = reactive and active power losses at the j branch with DG.

$K_{1},K_{2},{and\;K}_{3}$ = penalty factors.

The voltage stability index was used to find the multi-type DG, rating, and optimal location using PSO [142,252]. However, power demand was minimized by reducing power losses and increasing the voltage profile using the MOF optimization at the higher load level. The mathematical equation of MOF is given in Equation (16). The CLSF-based method was reported for placing a single DG capable of pre-specified dispatch into the energy grid [253]. Using that method does not provide the best solution. It further states that voltage magnitude is unsuitable for indicating the proximity to voltage collapse [134,254,255].

Moreover, the use of PLI, reactive power loss index (QLI), and voltage deviation index (VDI) are formulated in multi-objective indices (MOI), and line loading index (LLI) and short circuit index (SCI) are subjected to constraints as reported [256]. The target was to get the fuzzy and GA objective function, which challenges the uncertainty associated with weight factor selection. It was concluded that the performance of the methods is more accurate when compared to the weighted sum method, but the time in the computation was not considered. Furthermore, the problem is unconstrained with the most significant DG suitability index (DGSI) value, which gives the best location for DG placement. In contrast, the PLI and VSI are the input-driven forces for DGSI output. The optimization methods used for DG sizing and placement are AM and fuzzy logic, respectively, but economic factors were neglected [257,258].

(16) $$MOF=\sum_{i=1}^{nb}{\left(I_{bi}\right)}^{2}+K\sum_{i=1}^{Nb}{\left(V_{i}-V_{rated}\right)}^{2}$$

where *K* is the weighting factor, and $V_{rated}$ is the steady-state voltage magnitude.

Nouri presented a MOF, which contains the weighted sum of the line loss reduction index (ILRI), line transmission apparent power improvement index (LTAPII), and voltage profile improvement index (VPII) [259]. GA minimizes the MOF value after the optimum allocation of multiple DGs. The GA technique was used to identify the node for optimum DG placement, and the PSO method was used to optimize the sizing of DG [260]. The combined techniques GA-PSO perform better in terms of varying objective functions and optimized solutions than applied alone, but it takes a lot of time. Mathematically, the objective function is given in Equation (17). A new power stability index approach was reported to study the sensitive node close to instability, leading to voltage collapse when additional load is added [261]. The node with the higher value is selected for placement of DG [261]. For optimum DG rating, the branch current loss formula was used. The burden in computation is based on the analytical method, which was reduced by 50–60 % compared with the golden section of the search algorithm. The work is only considered single DG accommodation [262].

(17) $$f=minimize\left[\left(f_{1}+f_{2}K_{1}+f_{3}K_{2}\right)+\beta_{1}\sum_{j\;\epsilon \;N_{DG}}\left\{\max\left(V_{j}-V_{j}^{\max},0\right)+\max\left(V_{j}^{\min}-V_{j},0\right)\right\}+\beta_{2}\sum_{j\;\epsilon \;nb}\;\max\left\{\left|S_{j}\right|-\left|S_{j}^{\max},0\right|\right\}\right]$$

where $f_{1},f_{2}\;and\;f_{3}$ are the network power loss, voltage profile, and voltage stability index, respectively, $K_{1}$, $K_{2},\beta_{1}$ *and* ${\;\beta }_{2}$ are the coefficient of penalty, $V_{j}$ is the voltage magnitude, $V_{j}^{\max}$, $V_{j}^{\min}$ are the maximum and minimum voltage, $S_{j}^{\max}$ is the maximum apparent power ${S\;}_{j\;}$ is the apparent power and $N_{DG\;}$ is the total number of DG.

A PSO technique was presented to reduce MOF, such as lowering PLI, QPLI, and voltage profile improvement in the IEEE 30 bus system by incorporating DG. The method was reported to reduce power loss and improve bus voltage with DG [263]. A heuristic-iterative method of two nested calculation stages was reported [264]. At the external stage, some buses were selected using a clustering-based approach with normalized loss sensitivity factor and bus voltages. At the internal location, energy loss is the objective function of driving specific search techniques to decide the useable DG ratings set at the participant buses. The objective function is given in Equation (18).

Zareiegovar et al. [265] reported the PSO to minimize power loss, VSI, and improve voltage profile. PSO yielded a better result compared with combined GA-PSO, but only active power was supplied by DG and the ${PF}_{DG}$ was assumed to be 0.9. Moreover thermal limit constraint was neglected [265]. Improve Harris Hawks Optimization algorithms (HHO) were used for single and multi-objective tasks called IHHO and MOIHHO to place DG in RDS. The approach minimizes voltage deviation and power loss and increases VSI. The performance results were compared to bacterial foraging optimization algorithm (BFOA), backtracking search optimization algorithm (BSOA), TLBO, quasi-oppositional TLBO (QOTLBO), etc., regarding quality solutions in single and multi-objective functions IHHO and MOIHHO outperform them. In compared to other methods, IHHO and MOIHHO provide quality solutions [54]. A novel transient search optimization (TSO) was reported to minimize power loss and voltage deviation and enhance voltage profile with multiple placement of DG. The results revealed that TSO gives better results than GA, MOHHO, PSO, TLBO, and hybrid GAPSO [55].

The voltage stability index $\left(\frac{V}{V_{0}}\right)$ for a perfect three-phase system was given [266]. The positive sequence voltage ranking index (VRI) of $\left(\frac{V_{collapse}}{V_{no\;load}}\right)$ was presented [267]. VRI was used to identify the sensitive busin a balanced three-phase and unbalanced system network for optimum placement of capacitors and DGs. However, the capacity of DGs is increasing in three-phase unbalanced system networks that use iterative algorithms to reduce system losses and give better VSM performance. At the same time, the bus voltage limits were considered [[268], [269], [270], [271]]. It was concluded that for the enhancement of the system, there is a need to improve the algorithm's performance for the placement of DG.

(18) $$Minimize\;C_{X}=\propto \frac{F_{LX}}{F_{L}X^{base}}+\left(1-\alpha \right)\frac{F_{VX}}{F_{V}X^{base}}$$

$F_{LX}=$ Energy loss.

$F_{L}X^{base}=$ Energy loss without local generation.

$F_{V}X^{base}=$ Voltage profile without local generation.

$F_{VX}=$ Voltage profile function.

∝= Weighting factor.

Optimization of multiple wind energy-based DGs was reported using the DE algorithm [272]. The constraints in the line flow of the grid were considered. The voltage stability used an incremental voltage $\left(\frac{dv}{dp}\right)$ sensitivity approach for optimum selection of node in a sub-transmission system and the higher load was taken into consideration. Type 3 DG was used in the study to minimize transmission losses. The obtained result was compared with bare bones PSO (BBPSO) and multi-membered non-recombinative evolution strategy (MMNRES); DE give satisfactory solution [272].

In another study [273], the indices of the weakest bus and the line close to voltage collapse were normalized and merged. It was represented as the first fitness function (F1), and the second fitness function (F2) is the normalized status of branch loss reduction. PSO algorithm was used to reduce the weighted combination of F1 and F2. It was reported that there is no constant criterion for weight factor selection, and line load constraints were neglected. The result was compared to the grid selection method and AM, and PSO outperformed them in reducing loss, improving voltage profile, and enhancing line stability and loading factor [273]. Hassan et al. reported a binary PSO and shuffled frog leap (BPSO-SLFA) to reduce the system's single and multi-objective functions [274]. It was reported that the power loss was reduced with the optimum placement of DG, and voltage stability was improved. The proposed method was tested on the IEEE 33 and 69 bus systems, and the result shows better performance than hybrid Big Bang Big Crunch and grey wolf optimization (GWO) [274]. Evolutionary multi-objective optimization was used for the optimum allocation of DG to lower power loss, total cost, and GHE and enhance the system's reliability and voltage. The comparison of the proposed method gives better results than MOPSO, DAPSO, and AEPSO [275]. Multiobjective Lichtenberg (MOTA) and thermal exchange optimization (MOTEO) methods have been used for optimum allocation of DG and capacitors to minimize voltage deviation, power loss, and voltage stability improvement [167]. The result reveals that MOTEO performs better than MOTA. Therefore, authors suggested that MOTEO should be used in the future for reliability indices, energy not supplied, and annual cost [167].

## 7 Methods used in placement and sizing of DG for voltage stability improvement

This section reports the different methods for DG's location and placement for voltage stability improvement. The methods are the conventional and meta-heuristic algorithms (single and hybrid). Due to the continuous development of new algorithms, researchers have combined them with state-of-the-art algorithms to obtain optimum DG placement and sizing results. Convention methods usually suffer from local optimal, which results in less efficient solutions. The limitations of MINLP are the risk of the high dimensionality of the problem and the impossibility of considering nonlinear effects [276]. In large-scale power systems, CPF has been reported to be time-consuming [277]. Therefore, single metaheuristic algorithms have been used to overcome the drawbacks of conventional methods. However, some algorithms still stocked to the local optimal (i.e., they do not attain the global optimum solution to a problem). For example, VSA has been reported as not escaping from the local minimum point due to adaptive step size adjustment in VSA [278]. Also, ABC has been reported to have a slow and premature convergence rate [279].

Furthermore, hybridization algorithms come in place to overcome the limitation of convention and single algorithms for quality solutions to problems, but this method is complex when writing the program. The most objective function considered is power loss reduction. Table 4 illustrates different methods and objective functions considered by various authors, while Table 5 compares each method's advantages and disadvantages.

**Table 4:** Methods used for optimum placement of DG for voltage stability improvement.

Categories	Optimization methods	Objective function	IEEE test system	Contribution	Software used	References
Convention methods	MINLP	Loss reduction	33 and 69 bus systems	Multi-node modeling approach, convex envelopes, reducing the computational time and search space using the capacity planning model (CPM) and siting planning model (SPM) to improve MINLP.	MATLAB	[[280], [281], [282], [283]]
	CPF	Reducing power loss and improving voltage profile	85 bus systems	Maximization of system loadability with DG penetration	PSAT	[284]
	AM	Minimize the power loss of the system.	15 and 33-bus systems	Hybridization of the analytical method with OPF and a combined index based on the combination of active power loss index and VSI to select the node for DG allocation	MATLAB	[134,163,176,285]
	OPF	Loss reduction	41 bus system	Combined active and reactive OPF (A-R-OPF) was used for battery energy storage (BESS)	GAM and MATLAB	[286]
Single meta-heuristic algorithms	PSO	Minimizing power losses and voltage deviation	12, 30, 33, and 69 bus systems	Application of swarm-based algorithm for DG allocation	MATLAB	[273,287]
	TLBA	Reduction in power losses and voltage profile	69 and 119 bus systems	A mutation strategy was used to improve the basic TLBA for better global operation and to avoid premature convergence.	MATLAB	[78]
	SLPSO	Reducing the power loss and improving the network voltage profile	30 bus system	Social learning PSO was used for optimal sizing and allocation of DG	MATLAB	[288]
	WOA	Minimize system loss and yearly economic loss.	33 and 69 bus systems.	Optimized DG rating source to simultaneously minimize power loss and annual economic loss.	MATLAB	[206]
	SCA	Minimize the system power loss	15, 33, and 69 bus systems.	Application of population-based optimization for optimal location and sizing of DG.	MATLAB	[56]
	DA	Reduction in system real power losses	15, 33, and 69 bus systems	Application of swarm intelligence (DA) for loss reduction.	MATLAB	[289]
	ABC	Reduction in power loss and voltage drop	33 and 69 bus systems.	A swarm-based algorithm (ABC) was used for the CIGRE benchmark function to minimize multiple objective functions simultaneously.	MATLAB	[290]
	MMPO	Reduction in power loss and VSI	33 and 69 bus systems.	The predator strategy is used for possible variations in environmental and climate circumstances.	–	[291]
	GA, HSA	Reduction in power losses	33 bus system.	Comparison of an evolutional-based algorithm with swarm intelligence for optimal location and sizing of DG.	–	[292]
	VSA	Minimize the daily energy losses.	33 and 69 bus systems.	A master-slave strategy based on a discrete-continuous method was used in the master stage to improve the basic VSA.	MATLAB	[293]
Hybrid methods	LSF and SCA	Minimize power losses and improve voltage profile.	33 and 69 bus systems.	The search space was reduced using LSF, and the optimum location was archived using SCA.	MATLAB	[294]
	CBGA-VSA	Reduction in power loss source and VSI	33 and 69 bus systems.	Chu-Beasley GA was employed to solve the master stage, and VSA was used in the slave stage to form a hybrid (HCBGA-VSA) approach.	GAMS	[295]
	BPSO-SLFA	Reduction in active and	33 and 69 bus systems	Hybridization of two algorithms (BPSO-SLFA).	MATLAB	[274]
	SCA and chaos map theory	Reduction in power loss and improved voltage profile	33 and 69 bus systems.	An iterative chaotic map was incorporated to overcome the trapping optimal and low convergence during the exploration and exploitation of SCA.	MATLAB	[296]
	GA-PSO	Minimize network power losses, better voltage regulation, and improve the voltage stability.	33 and 69 bus systems.	The ability to achieve fast convergence in PSO was combined with GA to improve its global solution.	MATLAB	[260]
	HGPSO	Minimize the line active and reactive power losses and the deviation of bus voltages.	33 and 69 bus systems.	Hybridization of two population-based algorithms to obtain their advantages	–	[297]
	MOPSO	Power loss, voltage profile, pollution emissions, and DG cost	33 and 69 bus systems.	Global Pareto solution was used to update the Pareto optimal solution generated during the iteration	MATLAB	[298]
	AM-PSO	Node voltage and total active power loss	41 bus radial distribution system.	The application of AM-PSO is employed to optimally allocate DG.	MATLAB	[299]
	REPSO	Minimize active power loss.	33 bus system.	Rank evolution was hybridized with PSO to improve the particle movement toward better solutions and to obtain global and personal best in a simplifier manner.	MATLAB	[175]
	SPSO	Reduction in real power loss	69 bus system.	Selective PSO was used to improve basic PSO.	MATLAB	[300]
	SCA-SOCP	Reduction in power loss.	33 and 69 bus systems.	Hybridization of master-slave optimization was used. In the master stage, a discrete SCA was used, and SOCP was used in the slave stage.	MATLAB	[142]
	QOSIMBO-Q	To minimize power loss, improve voltage stability, and obtain better voltage regulation	33 and 69 bus systems.	Quasi-opposition-based learning (QOBL) was used to improve the limitation of a large number of iteration-facing Swine Influenza Model-Based Optimization with Quarantine (SIMBO-Q)	MATLAB	[301]
	MOEA/D	Reduction of load losses, reduction of load cost, and reduction of voltage drop in the network	33 and 69 bus systems.	Decomposition based on the evolutionary algorithm was used for multi-objective function.	–	[302]
	HPSO	Maximization of network load-ability	33 bus system.	Hybridization of discrete PSO to overcome premature convergence of basic PSO.	–	[229]

**Table 5:** **Comparison of methods used in DG placement and sizing** [81].

Optimization methods	Advantages	Disadvantages
Conventional	a. Easy implementation b. It does not have a convergence problem.	a. Lack of accuracy when the problem becomes complex. b. It is not robust.
Metaheuristic	a. It is more efficient than the conventional method. b. It works with continuous and discrete parameter c. Efficient in complex problem.	a. It is time-consuming due to repetition in fitness evaluation and the complexity of the problem. b. Possibility of trap to local optimal. c. It does not guarantee a high-quality solution when higher accuracy is required.
Hybrid	a. High efficiency than the conventional and metaheuristic. b. Less time-consuming. c. It gives an optimal global solution.	a. It is very complex.

## 8 Conclusion and future work

As the utility company faces the voltage instability problem, this review discusses some methods to identify weak buses and the impact of optimum DG placement to minimize losses that could lead to voltage collapse in the PS network. This paper presents the relevant aspects of DG, its definition, and its impact on voltage stability. The benefits of DG and its technologies on the DN were discussed. This paper also gives a detailed review of various DG technologies not presented in previous work. Also, the importance of DG on power loss minimization, voltage stability improvement, energy loss minimization, and multi-objective optimization were discussed. Multi-objective optimization gives a better approach to DG planning by implementing the best solution among many single-objective functions. Various algorithms and conventional methods used in the sizing and placement of DG for voltage profile improvement were also discussed, along with the comparison. A hybrid metaheuristic algorithm was recommended to give better loss reduction and adequately improve the voltage profile, leading to voltage stability. The approaches mentioned in this paper will help PS utility companies, engineers, researchers, and operators improve the system's operation to give a good voltage profile, minimize loss, and improve voltage stability. Future work in planning and integrating non-traditional DG in PS requires more work.

(1). Incorporating solar PV into the PS is essential to elevate the issues of voltage instability that could lead to voltage collapse. Solar PV is a stand-alone device that generates power from the sun. Therefore, there is a need for more research in this area so that consumers can afford the installation cost.
(2). Planning of DG should put modeling of different uncertain factors, e.g., solar, wind power generation, growth in future load, unstable market prices, and demand in an electric vehicle charging should be put into consideration to minimize investment cost and encourage electric vehicle users.
(3). Also, from various technologies of DG, more research is needed on fuel cells since it is another technique of DG so that voltage instability issues could be minimized when incorporated into PS networks.
(4). DG provides voltage support during the steady-state operation in distribution networks, which has been discussed. Reactive power provided by DG is limited due to the small size of DG, and priority is given to active power generation. However, other means of reactive power compensation coordinated with DG should be provided. More research is needed for frequency stability with DG, e.g., considering energy storage technologies to explore more DG potential.
(4). The hybrid method should be used to compare different types of DG with flexible alternating current transmission systems (FACTS) devices in future work.
(5). All the hybrid methods mentioned above combined two techniques, SCA-SOCP, HGPSO, and GA-PSO. More work is needed to combine three or more of these methods to form a robust optimization method for the sizing and placement of DG. Also, Table 4 shows that more authors used MATLAB software for DG integration. Therefore, more software should be used than MATLAB or a combination of two software to check which one has a better solution.
(6). Various authors have worked on DG placement for power and energy loss reduction and voltage profile improvement. Still, the reduction in harmonic pollution has not been presented, which requires more work.

## CRediT authorship contribution statement

**Samson Ademola Adegoke:** Writing – review & editing, Writing – original draft, Visualization, Validation, Methodology, Investigation, Formal analysis, Data curation, Conceptualization. **Yanxia Sun:** Writing – review & editing, Visualization, Validation, Supervision, Conceptualization, Funding acquisition. **Adesola Sunday Adegoke:** Writing – original draft, Visualization, Methodology, Validation, Conceptualization. **Damilola Ojeniyi:** Writing – original draft, Visualization, Validation, Methodology, Conceptualization.

## Ethics approval and consent to participate

This article does not contain any studies involving human or animal subjects.

## Ethics statement

Not applicable.

## Availability of data and materials

All data and materials used in this study are available within this article.

## Funding information

There is no funding available for this work.

## Declaration of competing interest

The authors declare that they have no known competing financial interests or personal relationships that could have appeared to influence the work reported in this paper.
